# Genome-Wide Identification and Comparative Profiling of MicroRNAs Reveal Flavonoid Biosynthesis in Two Contrasting Flower Color Cultivars of Tree Peony

**DOI:** 10.3389/fpls.2021.797799

**Published:** 2022-01-04

**Authors:** Xiaoning Luo, Sha Luo, Yaqi Fu, Chen Kong, Kai Wang, Daoyang Sun, Mengchen Li, Zhenguo Yan, Qianqian Shi, Yanlong Zhang

**Affiliations:** ^1^College of Landscape Architecture and Art, Northwest A&F University, Yangling, China; ^2^Academy of Agricultural Planning and Engineering, MARA, Beijing, China

**Keywords:** MicroRNA, tree peony, flavonoid biosynthesis, yellow pigmentation, regulatory mechanism

## Abstract

MicroRNA (miRNA)-mediated gene regulation is involved in various physiological processes in plants. Flower color is one of the vital ornamental traits of tree peony (*Paeonia suffruticosa* Andr.). However, the yellow-flowered tree peony cultivars are particularly rare. To elucidate the miRNA-mediated gene regulatory mechanism underlying yellow pigmentation in tree peony, we combined pigment assessment, miRNA identification, expression analysis, and gene functional verification in two contrasting flower color cultivars “High Noon” and “Roufurong.” Flavones/flavonols and anthocyanins were found to be the main contributors to the coloration of “High Noon” and “Roufurong” petals, respectively. Subsequently, miRNA analysis based on available genome data identified 9 differentially expressed miRNAs and 12 relevant target genes implicated in flavonoid biosynthesis. Their dynamic expression patterns determined the key role of mdm-miR156b-PsSPL2 module in yellow pigmentation of tree peony flowers. The sequence analysis and subcellular localization validated that PsSPL2 might function as a nuclear-localized transcription factor. Overexpression of *PsSPL2* in tobacco resulted in a decrease of anthocyanin content and down-regulation of *NtF3′H* and *NtDFR* transcripts. *PsSPL2*-silenced petals exhibited lighter yellow color, and the contents of THC, Ap, and Ch decreased significantly. Meanwhile, expression levels of *PsCHS*, *PsCHI*, and *PsF3H* were significantly decreased in the petals with *PsSPL2* silencing, while those of *PsF3′H* and *PsDFR* were remarkably increased. This study offers a novel insight into yellow pigmentation-related miRNA regulation network in tree peony, and further provides the valuable information on physiological changes during yellow coloring process of tree peony.

## Introduction

MicroRNAs (miRNAs) are endogenous non-coding RNAs with a length of 19–24 nucleotides, which play crucial roles in regulating plant growth and development and responding to environmental stimuli at the post-transcriptional level (Tang and Chu, 2017; Aydinoglu and Lucas, 2019). It is well established that miRNAs inhibit the expression of target genes by mRNA degradation, translation suppression, and DNA methylation through the imperfect sequence complementarity (Rogers and Chen, 2013; Li et al., 2016). Thus, it is credible to predict the target genes of miRNAs using bioinformatic analysis of related miRNA-mRNA complementary sequences. Transcription factors (TFs) occupy the major target genes of miRNAs, followed by nucleotide-binding proteins, leucine-rich repeat proteins, pathogen proteins, long non-coding RNAs, and other proteins (Bo and Wang, 2005; Tang and Chu, 2017).

In plants, miRNAs have been reported to participate in various biological activities, including flower development, leaf morphogenesis, signal conduction of hormones, transition of developmental timing, and responses to biotic and abiotic stresses (Jones-Rhoades et al., 2006; Pashkovskiy and Ryazansky, 2013). With the speedy development of sequencing technology in recent years, the deep sequencing of miRNAs has been widely performed in numerous plants, such as sweet orange (*Citrus sinensis* L. Osbeck) (Xu et al., 2010), safflower (*Carthamus tinctorius* L.) (Cao et al., 2013), rose (*Rosa* spp.) (Pei et al., 2013), Chinese white poplar (*Populus tomentosa* Carr.) (Tang et al., 2016), canna (*Canna indica* L.) (Roy et al., 2016), litchi (*Litchi chinensis* Sonn.) (Liu et al., 2017), and herbaceous peony (*Paeonia lactiflora* Pall.) (Zhao et al., 2017a). However, only few studies about the involvement of miRNAs in flower color formation have been reported. For instances, 109 miRNAs targeting 1,343 genes are identified to be differentially expressed in pale yellow-flowered and red-flowered canna. Amongst them, 5 miRNA families and 5 relevant target genes have been revealed to participate in phenylpropanoid and pigment metabolic processes (Roy et al., 2016). Similarly, 163 conserved and 28 novel miRNAs show differential transcription in red outer-petal and yellow inner-petal of herbaceous peony, and the regulation of miR156e-3p-targeted *squamosa promoter binding protein-like 1* (*SPL1*) makes a significant contribution to the yellow petal formation (Zhao et al., 2017b).

Tree peony (*Paeonia suffruticosa* Andr.), belonging to the section *Moutan* in the genus *Paeonia* of the family Paeoniaceae, is a kind of woody plant with great ornamental values (Li, 1999). Flower color is one of the most important ornamental traits of tree peony, and more than 2,000 cultivars with 9 major colors have been produced worldwide by conventional breeding (Gu et al., 2018; Yang et al., 2020b). The flowers of Chinese tree peony cultivars are usually white, pink, purple or red but lacking pure yellow. At present, the cultivation of yellow-flowered tree peony plants shows great prospects (Han et al., 2018). The chemical substances determining the flower colors mainly include flavonoids, carotenoids, and betalains (Zhou et al., 2009). To the best of our knowledge, the pigment composition of yellow flowers in plants is relatively complex, which is mainly related to flavonoids and carotenoids (Zhou et al., 2009). The types of flavonoids and carotenoids vary in different plant species. It has been found that flavonoids covering anthocyanins, flavones, and flavonols are the main pigments in tree peony flowers, except for the existence of chlorophylls in petals of a few green-flowered cultivars (Zhou et al., 2011b). Abundances of flavonoid components such as apigenin (Ap), kaempferol (Km), luteolin (Lu) glucosides, and chalcones have been detected in yellow-flowered tree peony cultivars (Li et al., 2009). Additionally, chalcones, tetrahydroxychalcone (THC), isosalipurposide (ISP), quercetin (Qu), Km, Ap, isorhamnetin (Is), and chrysoeriol (Ch) are the main flavonoid components in yellow flowers of *P. lutea* (Zhou et al., 2009). A lot of flavonoid synthesis-related structural genes and TFs have also been identified in tree peony, including *PsCHS1*, *PsCHI1*, *PsF3H1*, *PsDFR1*, *PsANS1*, *PlWDR3*, *PlWDR18*, *PlbHLH3*, and *PsMYB12* (Zhou et al., 2011a,b, 2014; Shi et al., 2015a,b, 2017; Gu et al., 2018). Overexpression of *PsCHI1* in tobacco (*Nicotiana tabacum*) significantly increases the content of flavones and flavonols, and decreases the content of anthocyanins, resulting in lighter flower color (Zhou et al., 2014). High expression levels of *THC2′GT*, *CHI*, and *FNS II* in flowers of *P. lutea* contribute to the coloration of yellow pigments (Shi et al., 2015b). With respect to TFs, PsMYB12 forms a complex with a bHLH TF and a WD40 protein to directly regulate the expression of *PsCHS*, thereby affecting the formation of petal blotch (Gu et al., 2018). Apart from MBW complex, additional TFs and regulatory genes have also been proposed to be involved in flavonoid biosynthesis, such as *SPL*s, *COP1*, *NAC*s, and *WRKY*s (Gou et al., 2011; Maier et al., 2013; Zhou et al., 2015; Li C. et al., 2020), while few findings have been reported in tree peony.

“High Noon,” a hybrid of *P. suffruticosa* × *P. lutea*, has uniform pure yellow petals and strong ecological adaptability. It has been widely planted in the main tree peony producing areas around the world. The study on flavonoid metabolism pathway in yellow petals of “High Noon” will help to understand the physiological process associated with yellow pigmentation of tree peony flowers. In the present study, the quantitative assessment of pigments comprising total anthocyanins, flavones/flavonols, chlorophylls, and carotenoids at five flowering stages of “High Noon” was conducted. The comparative transcriptome sequencing of miRNAs was performed using the tree peony genome as background. The dynamic expression patterns of candidate miRNAs and their corresponding target genes were analyzed. A mdm-miR156b-PsSPL2 module involved in flavonoid biosynthesis attracted our attention, and the regulatory function of *PsSPL2* was further explored and verified.

## Materials and Methods

### Plant Materials

**FIGURE 1:**
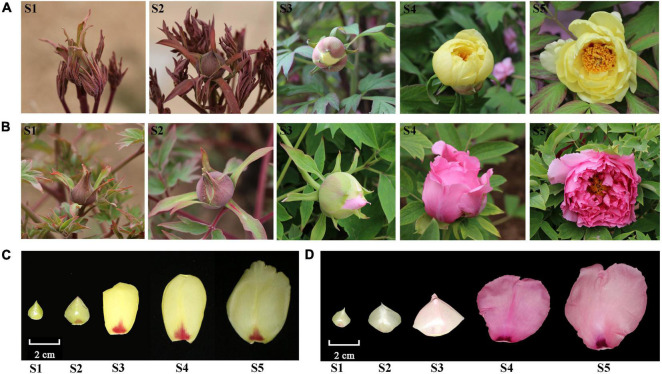
Flowers of two tree peony cultivars “High Noon” and “Roufurong” at five blooming stages. **(A,C)** “High Noon.”. **(B,D)** “Roufurong.” S1: Stage 1, unpigmented tight bud; S2: Stage 2, slightly pigmented soft bud; S3: Stage 3, initially opened flower; S4: Stage 4, half opened flower; S5: Stage 5, fully opened and pigmented flower with exposed anthers.

Tree peony cultivars “High Noon” (yellow flowers) and “Roufurong” (purple-red flowers) were planted in the Tree Peony Garden of Northwest A&F University, Shaanxi Province, China (34°26′ N, 108°07′ E). All plants were grown in the fields under natural light and moisture conditions. The petal samples of two cultivars at five blooming stages (S1, unpigmented tight bud; S2, slightly pigmented soft bud; S3, initially opened flower; S4, half opened flower; S5, fully opened and pigmented flower with exposed anthers) were gathered in April 2019 (Figure 1; Zhou et al., 2011b). The materials were immediately frozen in liquid nitrogen, and then stored at –80°C until further use.

### Measurement of Total Anthocyanins, Flavones/Flavonols, Chlorophylls, and Carotenoids

Total anthocyanins were extracted following the method of Fuleki and Francis (1968) with some modifications. Approximately 0.3 g of petals from “High Noon” (spots removed) and “Roufurong” at five blooming stages were ground to powder in liquid nitrogen and then rapidly homogenized with 6 mL of acidic methanol (1% HCl, v/v) in the dark at 4°C for 24 h. Subsequently, the supernatant was gathered under the centrifugation at 10,000 rpm for 10 min. The absorbance of total anthocyanins was measured with a Multiskan Spectrum (SP-Max 2300A2, Shanghai, China) from 200 to 850 nm. Total anthocyanin content was calculated using a molar absorbance coefficient of 26,900 (cyanidin-3-glucoside). Three biological replicates were used per sample, and the same was true for subsequent determination of anthocyanin content in tobacco petals.

Total flavones/flavonols were determined using a colorimetric method reported by Jia et al. (1999) with slight modifications. About 0.3 g of frozen petal powder for each sample was homogenized with 6 mL of methanol at 4°C in darkness for 24 h. After that, 0.3 mL of 5% NaNO_2_, 0.3 mL of 5% Al(NO_3_)_3_, and 4 mL of 4% NaOH were added into 5 mL of methanolic solution in turn, and kept for 6 min, 6 min, and 12 min, respectively. Finally, the mixture was diluted to 10 mL with methanol, and 0.2 mL of the mixture was transferred to a 96-well microplate. The absorbance of total flavones/flavonols was detected using the Multiskan Spectrum at 510 nm. Rutin served as the standard to establish a calibration curve. Total flavone/flavonol content was expressed as rutin equivalents.

Total chlorophyll and carotenoid contents were measured using the method described by Lichtenthaler and Buschmann (2001). About 0.3 g of petal powder was weighted and added into 6 mL of 80% acetone. After extraction at 4°C for 24 h, the absorbance of the extract at 470, 646, and 663 nm was determined, respectively.

### Library Construction, Sequencing, and Data Alignment

Total RNA from “High Noon” and “Roufurong” petals at S3 was extracted with Trizol Kit (Invitrogen, United States) following the manufacturer’s instructions. RNA purity was examined in an Agilent Bioanalyzer 2100 system (Agilent Technologies, United States) with RNA Nano 6000 Assay Kit. Then, total RNA was reversely transcribed into cDNA using SMARTer PCR cDNA Synthesis Kit (Takara, China). Two separated small RNA (sRNA) libraries with three biological replicates were constructed and subjected to sequencing on Illumina Hiseq 2500 platform.

Raw reads (which have been uploaded in the Sequence Read Archive of NCBI, PRJNA763093) were processed by eliminating the sequences with low quality and shorter than 18 nt or longer than 30 nt in length. Subsequently, clean reads were mapped to transfer RNA (tRNA) sequences in GtRNAdb^1^ (Chan and Lowe, 2009), ribosome RNA (rRNA) sequences in Silva^2^ (Quast et al., 2013), repetitive sequences in Repbase^3^ (Bao et al., 2015), and small nuclear RNA (snRNA) and small nucleolar RNA (snoRNA) sequences in Rfam^4^ (Nawrocki et al., 2015). The non-coding RNAs were removed by Bowtie software (Langmead, 2010). Then, the merged unique sequences were also aligned to tree peony reference genome^5^ using miRdeep^6^. Transcript Per Million (TPM) algorithm was applied to normalize and calculate miRNA expression (Fahlgren et al., 2007). Differentially expressed miRNAs (DEMs) were identified using DESeq R package (Anders et al., 2013). The criteria for DEMs were established as FDR ≤ 0.01 and | log2(fold change)| ≥ 1.

### Identification of Known and Novel MicroRNAs

To identify the known miRNAs, the unique sequences were mapped against the miRbase 22^7^ using a Basic Local Alignment Search Tool (BLASTn) (Kozomara and Griffiths-Jones, 2014). To identify the novel miRNAs, the remaining miRNA sequences were blasted against tree peony genome and the matched sequences were predicted for fold-back structure by Mfold program (Zuker, 2003). The sequences that have stem-loop precursors were considered as candidate novel miRNAs. After that, miRNA* sequences (complementary to miRNA in the precursor molecule) were searched in miRNA libraries, and those sequences with miRNA-miRNA* duplexes were deemed as novel miRNAs.

### Prediction and Annotation of MicroRNA Target Genes

TargetFinder software was used for predicting the target genes of miRNAs with default parameters (Bo and Wang, 2005). The function and annotation of putative target genes were analyzed by the following databases: NCBI non-redundant protein sequences (Nr)^8^, Protein family (Pfam)^9^, Swiss-Prot^10^, Gene Ontology (GO)^11^, and Kyoto Encyclopedia of Genes and Genomes (KEGG)^12^.

### Quantitative Real-Time Polymerase Chain Reaction Verification of Sequencing Data

Small RNA used for miRNA verification was extracted by miRcute miRNA Isolation Kit (Tiangen Biotech, China), and first-strand cDNA fragments were produced using miRcute Plus miRNA First-Strand cDNA Synthesis Kit (Tiangen Biotech, China). Total RNA used for target gene verification was extracted with RNAprep Pure Plant Plus Kit (Polysaccharides and Polyphenolics-rich) (Tiangen Biotech, China), and the reverse-transcription was performed through PrimeScript™ RT reagent Kit (TaKaRa, China). Then, quantitative real-time polymerase chain reaction (qRT-PCR) experiments were performed using TB Green TaKaRa Premix Ex Taq™ II (TaKaRa, China) according to the manufacturer’s specification on an ABI Prism 7500 Sequence Detector (Applied Biosystems, United States). The reaction was carried out under the following procedure: denaturation at 95°C for 15 s and 45 cycles of amplification (95°C for 5 s, 58°C for 30 s, and 72°C for 31 s). *Ubiquitin* and *U6* genes were used as internal references for expression normalization of target genes and miRNAs, respectively. Relative expression levels were calculated by 2^–ΔΔCT^ method. The primers used for expression assessment are listed in Supplementary Table 1. Three biological replicates were used for each qRT-PCR assay.

### Correlation Analysis

All data were expressed as the means ± SDs. Statistical analyses of pigment contents and gene expression levels were performed using Student’s *t*-test embedded in Excel 2013 software. Pearson’s correlation coefficients (*R* values) and the established heatmap were analyzed by *R* scripts.

### Sequence and Subcellular Localization Analyses

Based on the tree peony genome sequence resource, specific primers were designed to obtain the sequence of *PsSPL2* (Supplementary Table 1). The cDNA was synthesized using total RNA of the petals from “High Noon” at five blooming stages. Neighbor-joining method was used for constructing phylogenetic tree from evolutionary distance data by MEGA 6. Multiple sequence alignment was conducted using DNAMAN. The open reading frame (ORF) region of *PsSPL2* without the termination codon was cloned into the pCAMBIA1302-GFP vector, and in-fusion cloning primers were listed in Supplementary Table 1. Subsequently, the recombinant plasmid was transformed into *Agrobacterium tumefaciens* strain GV3101 through freeze-thaw method. The positive colonies were selected and incubated at 28°C to OD_600_ of 0.3 with the culture medium containing kanamycin. The *Agrobacteria* containing the target plasmids were resuspended in equal volume of infiltration buffer (10 mM MES, 10 mM MgCl_2_, and 100 mM acetosyringone) and stationarily cultured for 4–6 h at room temperature. The onion epidermis was immersed in a sterile dark environment for 6–12 h and cultured on MS solid medium for 3–4 days. Finally, the fluorescence was observed under a Nikon C2-ER confocal laser scanning microscope (Nikon, Japan). All transient expression assays were repeated three times.

### Overexpressing of *PsSPL2* in Tobacco

The tobacco (*N. tabacum*) leaf disk transformation was performed using *A. tumefaciens* strain GV3101 bearing pCAMBIA1302-*PsSPL2* plasmid following the previously described methods (Horsch et al., 1985). Transgenic tobacco lines were generated through the selection of kanamycin. Flowers of the second-generation transgenic plants were used for quantification of anthocyanin and gene expression. All gene-specific primers are shown in Supplementary Table 1.

### Silencing of *PsSPL2* in Tree Peony

Silencing of *PsSPL2* was conducted using a virus-induced gene silencing (VIGS) approach (Dai et al., 2012). Firstly, a 377-bp fragment at the 3′ end of *PsSPL2* cDNA was PCR-amplified for construction of the VIGS vector, and the gene-specific primers are listed in Supplementary Table 1. Next, the process of transformation and culture of *Agrobacterium* were the same as that of subcellular localization. When the final OD_600_ reached up to 1, mixture of cultures containing an equal ratio (v/v) of TRV1 and TRV2, TRV1 and TRV2-*PsSPL2*, were used for the empty vector control, and TRV-*PsSPL2* experiment, respectively. Before vacuum infiltration, the mixture was cultured for 4 h at room temperature in the dark. Petals of “High Noon” at S3 were collected, and the discs with 1.2 cm diameter were excised from the center of the petals using a hole punch. Ultimately, vacuum infiltration was conducted by soaking petal discs in the bacterial solution under a vacuum at 0.7 MPa. After vacuuming, petal discs were washed with deionized water and stored at 4°C for 2 days, followed by an equilibrium procedure at room temperature for one day before observation. The flavonoids were detected using a high-performance liquid chromatography (HPLC) method (Li et al., 2009). The qRT-PCR procedure was the same as described previously, and the specific primers were listed in Supplementary Table 1.

## Results

### Quantification of Pigments in Two Contrasting Flower Color Cultivars of Tree Peony

**FIGURE 2:**
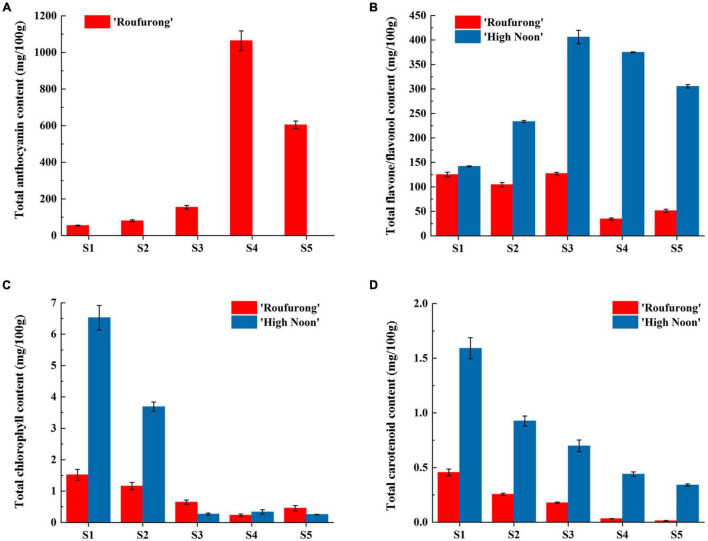
Total anthocyanin **(A)**, flavone/flavonol **(B)**, chlorophyll **(C)**, and carotenoid **(D)** contents of “High Noon” and “Roufurong.” All the measurements were performed in three biological and three technical replicates. Error bars represent standard deviation of biological and technical replicates.

Total anthocyanin, flavone/flavonol, chlorophyll, and carotenoid contents from petals at five flowering stages of “High Noon” and “Roufurong” were measured. As expected, no anthocyanin was detected in “High Noon” petals while total anthocyanin content in “Roufurong” increased first and then decreased, and reached a peak at S4 (Figure 2A). In contrast to total anthocyanins, total flavone/flavonol content of “High Noon” was significantly higher than that of “Roufurong,” reaching the maximum level at S3, indicating that flavone/flavonol might be responsible for yellow formation of “High Noon” petals (Figure 2B). Total chlorophyll and carotenoid contents in “High Noon” and “Roufurong” were both raised to the highest at S1 and then decreased as the flowers blooming (Figures 2C,D). However, in general, the contents of total chlorophyll and carotenoid in the two cultivars were much lower than those of total anthocyanin and flavone/flavonol, suggesting that chlorophylls and carotenoids possibly imposed a very weak effect on the pigment coloration of “High Noon” and “Roufurong” petals. Thus, we focused on the difference of flavonoid metabolism in the two contrasting flower color cultivars of tree peony, whose petals at S3 were selected for subsequent deep sequencing of sRNAs.

### Identification of Known and Novel MicroRNAs

A total of 89,042,099 clean reads with high Q30 (>99%) were screened (Supplementary Table 2), indicating that the deep sequencing data were reliable. After filtering out rRNAs, tRNAs, snRNAs, snoRNAs, and other repeats, the remaining reads were further mapped to the tree peony reference genome, and the percentage of mapped reads in each library ranged from 35.57 to 56.51% (Supplementary Table 3). Next, 5 known miRNAs and 320 novel miRNAs were identified (Supplementary Table 4). The known miRNAs were 19–24 nt in length, but no known miRNA of 22 nt or 23 nt has been identified (Supplementary Figure 1A). The length distribution of novel miRNAs ranged from 18 to 24 nt, with the length of 21 nt miRNAs being the most, followed by 24 nt (Supplementary Figure 1B). The first base bias analysis was also performed on all miRNAs, and the results showed that uridine was exactly one of the most common nucleotides (Supplementary Figure 2A). Additionally, the base bias analysis of each miRNA sequence site revealed that each site had a stronger preference for uridine and adenine (Supplementary Figure 2B).

### Analysis of Differentially Expressed MicroRNAs and Their Target Genes

Since miRNAs function by regulating the expression of corresponding target genes or inhibiting their translation, the identification of target genes are important parts of understanding the functions of miRNAs. Totally, 4,771 target genes for 294 miRNAs in tree peony were predicted (Supplementary Table 5), and annotated information was obtained for 4,532 out of 4,771 target genes (Supplementary Table 6). Among them, 3,475 targets returned significant blast hits against the NR database with 619 annotated targets possessed top hits to genes from *Vitis vinifera* (Supplementary Figure 3), indicating that the annotated targets of tree peony are highly similar to those of grape. This finding further proved the credibility of the sequencing data.

Totally, 165 DEMs were screened out, of which 56 were up-regulated and 109 were down-regulated in “High Noon” petals (Supplementary Figures 4A,B). The potential target genes of DEMs were predicted using mature sequences. Eventually, 3,009 target genes were characterized and annotated against 5 databases including GO (1,444), KEGG (1,091), NR (2,350), Pfam (1,910), and Swiss-Prot (1,794). GO analysis was performed to further investigate the function of miRNAs, and the putative targets of DEMs were clustered into three GO categories (biological process, molecular function, and cellular component) including 51 subcategories. Compared with other two categories, more targets fell under biological process, including three major subcategories metabolic process, cellular process, and single-organism process (Supplementary Figure 5A). Furthermore, KEGG enrichment results revealed that phenylpropanoid biosynthesis was one of the top five significantly enriched pathways (Supplementary Figure 5B).

### Analysis of Differentially Expressed MicroRNA-Target Gene Pairs Involved in Yellow Pigmentation

Based on the function prediction, 12 target genes underlying yellow pigmentation were selected, including 2 *PsMYB*s, 3 *PsSPL*s, 2 *PsNAC*s, 2 *PsWRKY*s, 1 flavonol 5-O-glucosyltransferase gene (*PsF5GT*), 1 chalcone isomerase gene (*PsCHI*), and 1 flavonoid 3-O-glucosyltransferase gene (*PsF3GT*). The corresponding miRNAs and cleavage sites are listed in Table 1. One miRNA may target one or more target genes. For examples, novel_miR_196 only targeted *PsF5GT* (psu.G.00004158) and novel_miR_138 targeted *PsCHI* (psu.G.00026462), while mdm-miR156b targeted three *PsSPL*s (psu.G.00031695, psu.G.00001221, and psu.G.00034722) simultaneously, and novel_miR_186 targeted both *PsMYB2* (psu. G. 00021962) and *PsWRKY1* (psu.G.00003037). It suggests that miRNAs might play a complex regulatory role in the coloration of tree peony flowers by inimitably regulating their target genes.

**TABLE 1:** The candidate DEM-target gene pairs involved in yellow pigmentation of tree peony.

Category	Target gene	miRNA	miRNA_seq	Cleavage site
*PsMYB1*	psu.G.00032738	novel_miR_260	CAUGUACUAUGAAUUGAGG	3′ GGAGUUAAGUAUCAUGUAC 5′
				|||:::||||| ||| :|||
				5′ CCUUGGUUCAU-GUAUAUG 3′
*PsMYB2*	psu.G.00021962	novel_miR_186	AUCUUCAGUGCUAAUGUCUGG	3′ GGUCUGUAAUCGUGACUUCUA 5′
				|| |:||||||:|||||||
				5′ CCUGUUAUUAGCGCUGAAGAC 3′
*PsSPL1*	psu.G.00031695	mdm-miR156b	UGACAGAAGAGAGUGAGCAC	3′ CACGAGUGAGAGAAGACAGU 5′
				|||||| |||||||||||||
				5′ GUGCUCUCUCUCUUCUGUCA 3′
*PsSPL2*	psu.G.00001221	mdm-miR156b	UGACAGAAGAGAGUGAGCAC	3′ CACGAGUGAGAGAAGACAGU 5′
				|||||| |||||||||||||
				5′ GUGCUCUCUCUCUUCUGUCA 3′
*PsSPL3*	psu.G.00034722	mdm-miR156b	UGACAGAAGAGAGUGAGCAC	3′ CACGAGUGAGAGAAGACAGU 5′
				|||||| |||||||||||||
				5′ GUGCUCUCUCUCUUCUGUCA 3′
*PsNAC1*	psu.G.00032930	novel_miR_101	UAUAAAACUGUUGAAAAUG	3′ GUAAAAGUUGUCAAAAUAU 5′
				:|| || :|||||||| |||
				5′ UAUAUUUAACAGUUUCAUA 3′
*PsNAC2*	psu.G.00017706	novel_miR_29	UCCCCUGCAUCUCCACCG	3′ GCCACCUCUACGUCCCCU 5′
				:|||| ||||| ||||||
				5′ UGGUGAAGAUGAAGGGGA 3′
*PsWRKY1*	psu.G.00003037	novel_miR_186	AUCUUCAGUGCUAAUGUCUGG	3′ GGUCUGUAAUCGUGACUUCUA 5′
				:| | :||||:||||||||||
				5′ UCUGGCAUUGGCACUGAAGAG 3′
*PsWRKY2*	psu.G.00018110	novel_miR_21	UUUAAGGGAUUUUAAAACAUC	3′ CUACA-AAAUUUUAGGGAAUUU 5′
				|||| |||||||| :| :||||
				5′ AAUGUGUUUAAAAUUCUUUAAU 3′
*PsF5GT*	psu.G.00004158	novel_miR_196	AUUGAUCCGACUUAAACGA	3′ AGCAAA-UUCAGCCUAGUUA 5′
				|| | |||| :||||||||
				5′ GAGUCUGAAGUUGGAUCAAU 3′
*PsCHI*	psu.G.00026462	novel_miR_138	AGGAAGAAAGUAGUAGAUGA	3′ AGUAGAUGAUGAAAGAAGGA 5′
				||||| ||| :||||||||
				5′ UCAUC-ACUGUUUUCUUCCC 3′
*PsF3GT*	psu.G.00030357	novel_miR_165	UUCCUGGAUUUGGUUCUCGCC	3′ CCGCUCUUGGUUUAGGUCCUU 5′
				| | ||| |||| ||||||||
				5′ GACCAGACCCAAUUCCAGGAA 3′

*DEMs were screened based on DEM set “Roufurong”_vs_“High Noon.”*

### Expression Patterns of Candidate Differentially Expressed MicroRNA-Target Gene Pairs by Quantitative Real-Time Polymerase Chain Reaction

To validate the reliability of sequencing data, 12 miRNAs were selected randomly to perform qRT-PCR on the basis of their pre-miRNA sequences (Supplementary Figure 6). As a result, expression levels of these miRNAs were basically consistent with those of deep sequencing data.

**FIGURE 3:**
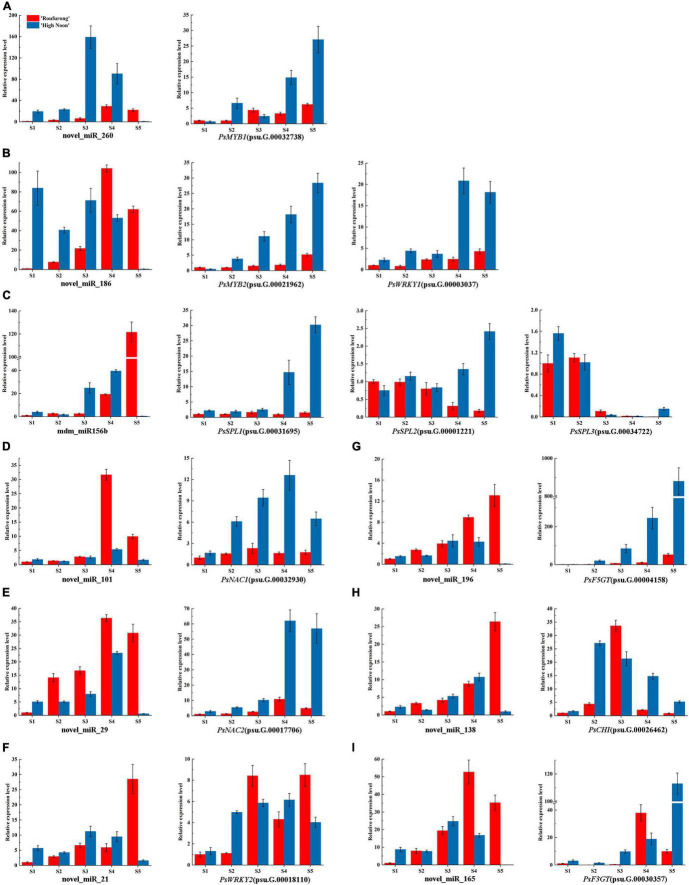
Dynamic expression patterns of candidate miRNAs and their corresponding target genes at five blooming stages in “High Noon” and “Roufurong.” **(A)** novel_miR_260 and its target gene *PsMYB1* (psu.G.00032738). **(B)** novel_miR_186 and its target genes *PsMYB2* (psu.G.00021962) and *PsWRKY1* (psu.G.00003037). **(C)** mdm_miR156b and its target genes *PsSPL1* (psu.G.00031695), *PsSPL2* (psu.G.00001221), and *PsSPL3* (psu.G.00034722). **(D)** novel_miR_101 and its target gene *PsNAC1* (psu.G.00032930). **(E)** novel_miR_29 and its target gene *PsNAC2* (psu.G.00017706). **(F)** novel_miR_21 and its target gene *PsWRKY2* (psu.G.00018110). **(G)** novel_miR_196 and its target gene *PsF5GT* (psu.G.00004158). **(H)** novel_miR_138 and its target gene *PsCHI* (psu.G.00026462). **(I)** novel_miR_165 and its target gene *PsF3GT* (psu.G.00030357). S1: Stage 1, unpigmented tight bud; S2: Stage 2, slightly pigmented soft bud; S3: Stage 3, initially opened flower; S4: Stage 4, half opened flower; S5: Stage 5, fully opened and pigmented flower with exposed anthers.

To further clarify the roles of miRNAs in tree peony yellow pigmentation, the dynamic expression patterns of 9 candidate miRNAs and their corresponding target genes at five blooming stages were detected (Figure 3). As far as miRNAs targeting TFs, expression levels of novel_miR_260 in “High Noon” were increased and peaked at S3 before decreasing, while its corresponding target gene *PsMYB1* showed a different expression pattern with the highest expression at S5. Expression levels of novel_miR_186 in “Roufurong” increased gradually and peaked at S4, whereas those of *PsMYB2* and *PsWRKY1* showed a slightly increasing trend throughout the blooming stages. The mdm_miR156b displayed a continuous increasing pattern in “Roufurong.” On the contrary, transcript levels of *PsSPL2* and *PsSPL3* presented a general downward trend. In “High Noon,” expression levels of mdm_miR156b decreased slightly from S1 to S2, then increased until the highest value at S4, and finally decreased sharply at S5. Conversely, expression levels of *PsSPL2* firstly increased from S1 to S2 and slightly decreased at S3, followed by a continuous increase until a peak at S5. *PsNAC1* and *PsNAC2*, as the target genes of novel_miR_101 and novel_miR_29, respectively, shared a similar expression pattern in two contrasting flower color cultivars of tree peony. Their abundances increased early and peaked at S4 before declining. Expression profiles of novel_miR21 and its target gene *PsWRKY2* in “High Noon” were also similar, with an initial increase followed by a decrease except that the level of novel_miR21 at S1 was slightly higher than that at S2. In addition to miRNAs targeting TFs, expression profiles of novel_miR_196 and novel_miR_165 targeting structural genes *PsF5GT* and *PsF3GT*, respectively, in “High Noon” were almost unexpressed at S5, while transcript abundances of *PsF5GT* and *PsF3GT* continued to elevate throughout the blooming process, and sharply peaked at S5. Finally, the expression of novel_miR_138 increased steadily during five blooming stages of “Roufurong,” while its target gene *PsCHI* exhibited no significant relationship. In “High Noon,” the transcription of novel_miR_138 increased erratically up to the highest at S4, and *PsCHI* showed an opposite trend. These results exhibited the negative relationship between the expression patterns of miRNAs and their corresponding target genes, which might be involved in the flavonoid biosynthesis pathway.

**FIGURE 4:**
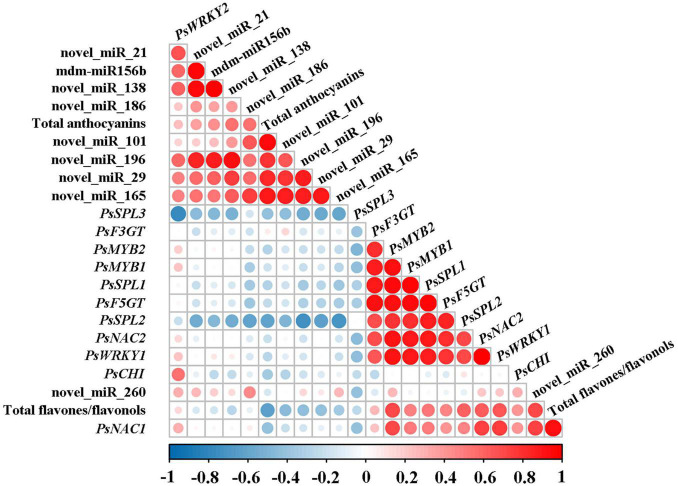
Correlation analysis between the expression of miRNAs and corresponding target genes and the accumulation of total anthocyanins and total flavones/flavonols. Pearson’s correlation coefficients for the data were analyzed using *R* scripts.

On the basis of the results above, the correlation analysis between the expression of miRNAs and corresponding target genes and the accumulation of total anthocyanins and total flavones/flavonols was carried out. As shown in Figure 4, total anthocyanin content showed a significant negative correlation with the expression of *PsSPL2* and *PsSPL3*, whereas total flavone/flavonol content presented a significant positive correlation with the expression of *PsNAC1*, *PsMYB2*, *PsSPL2*, *PsNAC2*, and *PsWRKY1*. It is worth noting that only *PsSPL2* was significantly and negatively correlated with total anthocyanins, but positively correlated with total flavones/flavonols. Moreover, expression pattern of *PsSPL2* was perfectly complementary to that of mdm-miR156b. Thus, *PsSPL2* acted as a bridge connecting mdm-miR156b and pigments, indicating that mdm-miR156b might affect the accumulation of anthocyanins and flavones/flavonols by regulating the expression of *PsSPL2*.

### Isolation and Subcellular Localization of *PsSPL2*

**FIGURE 5:**
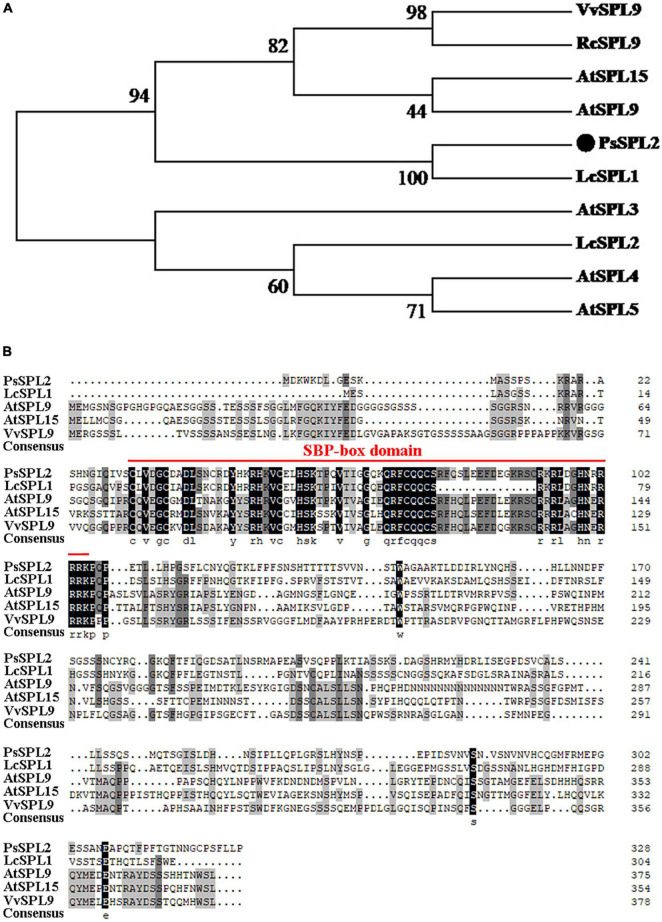
Phylogenetic analysis of PsSPL2 and its homologous proteins. **(A)** Phylogenetic analysis of PsSPL2 with other species. **(B)** Multiple alignments of deduced amino acid sequences of PsSPL2 with other species. NCBI accessions: VvSPL9 (NP_001267898.1), RcSPL9 (XP_015582800.1), AtSPL15 (NP_191351.1), AtSPL9 (NP_181749.1), LcSPL1 (AQM55950.1), AtSPL3 (NP_565771.1), LcSPL2 (AQM55951.1), AtSPL4 (NP_175723.1), AtSPL5 (NP_188145.1).

**FIGURE 6:**
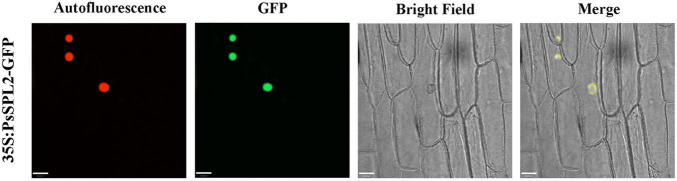
Subcellular localization analysis of PsSPL2 fusion protein in onion epidermal cells.

To investigate the function of *PsSPL2*, the full-length sequence of *PsSPL2* was isolated and characterized. Phylogenetic analysis with other species showed that PsSPL2 was attached to LcSPL1 (Figure 5A), which was reported to interact with the anthocyanin regulatory gene *LcMYB1* as a target gene of miR156a in *L. chinensis* (Liu et al., 2017). Compared with other SPL proteins, PsSPL2 and LcSPL1 were clustered together with AtSPL9, AtSPL15, RcSPL9, and VvSPL9, of which AtSPL9 has been identified to inhibit the accumulation of anthocyanin by imparing the stability of MBW complex in Arabidopsis (Gou et al., 2011). Additionally, PsSPL2 contained a typical SBP domain consisting of 79 amino acid residues (Figure 5B). The subcellular localization of *PsSPL2* was observed specifically in the nucleus of onion epidermal cells (Figure 6), suggesting that PsSPL2 probably function as a TF in regulating flavonoid biosynthesis.

### Overexpressing of *PsSPL2* in Tobacco

**FIGURE 7:**
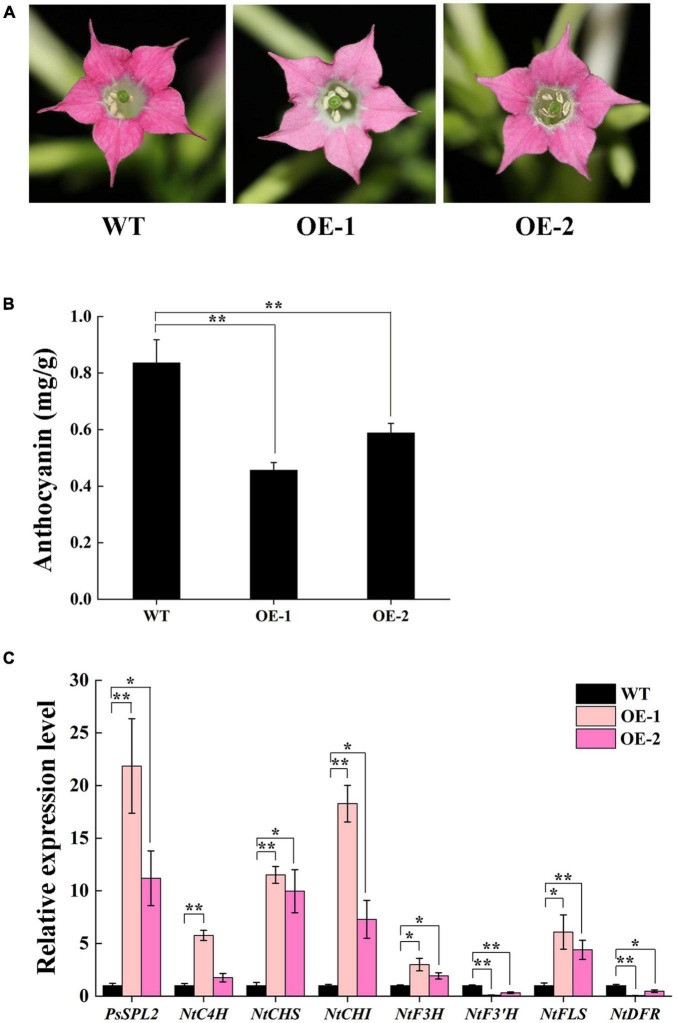
Overexpressing of *PsSPL2* in tobacco. **(A)** Flowers from wild-type (WT) and transgenic tobacco plants (OE-1 and OE-2). **(B)** Anthocyanin contents in petals of WT and transgenic tobacco plants. **(C)** Expression patterns of endogenous flavonoid biosynthetic genes in the petals of WT and transgenic tobacco plants. Error bars represent standard error of the mean from three biological replicates. **P < 0.01, *P < 0.05.

To characterize the function of *PsSPL2* in flavonoid biosynthesis process, we generated two transgenic tobacco lines (OE-1 and OE-2) overexpressing *PsSPL2*. Compared to the rosy red color of wild-type (WT), OE-1 and OE-2 displayed different degrees of fading, especially OE-1 (Figure 7A). Consistent with this, anthocyanin contents of both OE-1 and OE-2 were significantly lower than that of WT, and the anthocyanin content of OE-1 was also lower than that of OE-2 (Figure 7B). Additionally, *PsSPL2* and endogenous structural genes in flavonoid synthesis pathway of tobacco were also detected (Figure 7C). Expression levels of *PsSPL2* in OE-1 and OE-2 were significantly higher than those in WT. Similarly, the transcription of *NtC4H*, *NtCHS*, *NtCHI*, *NtF3H*, and *NtFLS* in OE-1 increased significantly compared to WT, whilst *NtF3′H* and *NtDFR* presented significantly lower transcript abundances than WT. Taken together, the gene expression patterns in OE-1 and OE-2 were correlated with their phenotypes and anthocyanin quantification results. These results suggest that *PsSPL2* possibly play a critical role in the accumulation of anthocyanin through negative regulation of some structural genes such as *F3′H* and *DFR.*

### Silencing of *PsSPL2* in Tree Peony

**FIGURE 8:**
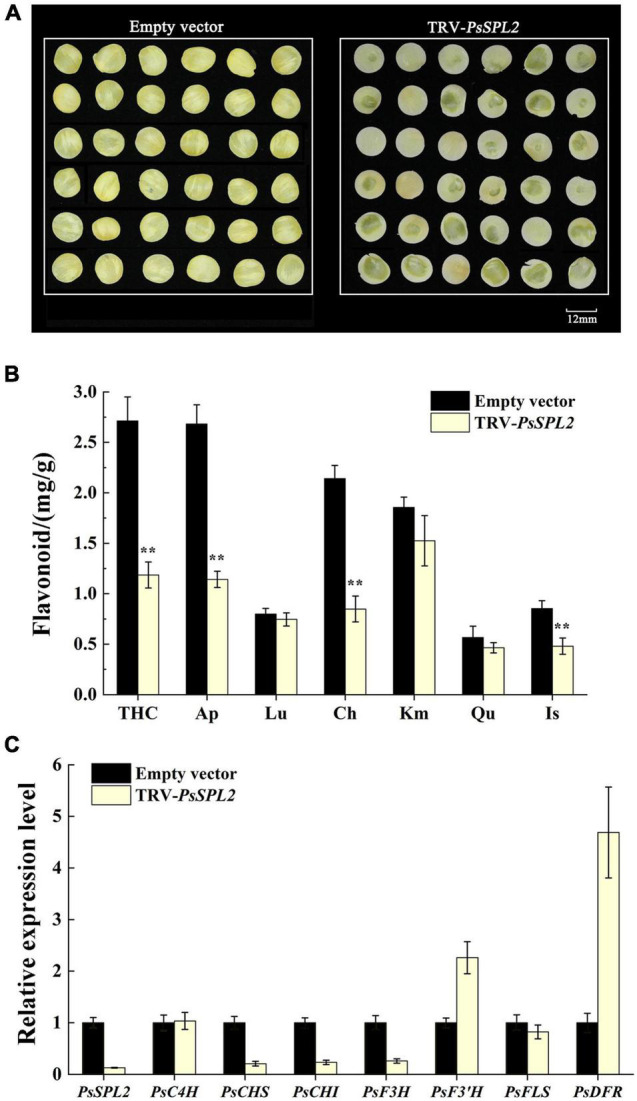
Silencing of *PsSPL2* by virus-induced gene silencing (VIGS) in petal discs of “High Noon.” **(A)** The phenotypes of the petal discs with *Agrobacterium* bearing a TRV empty vector and TRV-*PsSPL2*, respectively. **(B)** Flavonoid contents in petal discs with *Agrobacterium* bearing a TRV empty vector and TRV-*PsSPL2*, respectively. **(C)** The relative expression levels of *PsSPL2* and corresponding structural genes by quantitative RT-PCR in petal discs with *Agrobacterium* bearing a TRV empty vector and TRV-*PsSPL2*, respectively. Error bars represent standard error of the mean from three biological replicates. **P < 0.01.

To further identify the potential role of *PsSPL2* underlying yellow pigmentation in tree peony, we observed the phenotypes of *PsSPL2*-silenced petal discs using a VIGS approach. As shown in Figure 8A, the color of *PsSPL2*-silenced petal discs was significantly lighter than that of empty vector control. Moreover, the contents of flavonoid such as THC, Ap, Lu, Ch, Km, Qu, and Is were detected, and the results showed that the content of individual component of *PsSPL2*-silenced petal discs decreased in varying degrees (Figure 8B). In particular, the contents of THC, Ap, and Ch were significantly reduced compared to those of the empty vector control, suggesting that THC, Ap, and Ch contribute to the yellow pigmentation of tree peony. Correspondingly, transcript abundances of *PsSPL2* and structural genes that regulate the synthesis of these flavonoids were also examined (Figure 8C). As a result, expression levels of *PsSPL2*, *PsCHS*, *PsCHI*, and *PsF3H* decreased in *PsSPL2*-silenced petal discs, while expression levels of *PsF3′H*, and *PsDFR* increased. The significant down-regulation of *PsCHS*, *PsCHI*, and *PsF3H* might be responsible for the decreased production of THC, Ap, and Ch. In contrast, it is worth noting that the expression of *PsDFR* was significantly up-regulated, indicating that there may be a direct regulatory relationship between *PsSPL2* and *PsDFR*.

## Discussion

The vibrant and attractive flower colors of tree peony have received considerable attention, and many genes involved in its pigment coloring have been reported. However, the roles of miRNAs in the coloring process of yellow-flowered tree peony cultivars remain to be unclear. In our study, the pigment detection, sRNA sequencing, expression analysis, and gene functional characterization were performed to understand the mechanisms of yellow pigmentation in tree peony. Given that the content of total chlorophylls and carotenoids in petals of “High Noon” and “Roufurong” was minimal compared to total flavones/flavonols and anthocyanins (Figure 2), we believed that flavones/flavonols and anthocyanins were the main contributors to the coloration of “High Noon” and “Roufurong” petals, respectively. Similarly, low levels of carotenoids in *Canna* cultivars, Tropical sunrise (TS) and Red president (RP), indicate that carotenoids may not be the major source of pigments in floral coloring of *Canna* (Roy et al., 2016). Previous studies also reported that the petal coloring pigments of tree peony are flavonoids, including anthocyanins, flavonols, and chalcones (Wang et al., 2001a,b; Li et al., 2009; Zhou et al., 2009; Yang et al., 2020a). There were 39 flavonoids covering 5 anthocyanins, 12 flavones, 21 flavonols, and one chalcone in 8 different color series from white to red to yellow in tree peony (Li et al., 2009; Fan et al., 2012). Chalcone 2′G is the main flavonoid component in yellow flowers of *P. lutea* while anthocyanin is undetected. However, Pn and Cy type anthocyanins determine the color tone of purple-red flowers of *P. lutea* (Shi et al., 2015b). There is also a big difference in the constitutive levels of flavonoids in the petals between “High Noon” and “Roufurong,” which is helpful to investigate the flavonoid biosynthetic pathway.

As far as we know, previous studies on the transcriptome of tree peony were based on mRNA sequencing at the transcriptional level (Shi et al., 2015b; Gao et al., 2016; Luo et al., 2017), whereas the present study firstly combined sRNA sequencing with genomic data at the post-transcriptional level. On this basis, a total of 5 known miRNAs and 320 novel miRNAs were identified in “High Noon” and “Roufurong” floral tissues. Differing from a large number of known miRNAs in other species such as *P. lactiflora* (Zhao et al., 2017a), *L. chinensis* (Liu et al., 2017), and *N. tabacum* (Jin et al., 2020), the number of novel miRNAs identified in our study was much higher than that of known miRNAs. It may be attributed to the specificity of tree peony genome.

According to the functional prediction, 12 target genes underlying yellow pigmentation and 9 related miRNAs were further selected. These target genes consisted of four types of TF genes (*MYB*, *WRKY*, *NAC*, and *SPL*) and three structural genes (*F5GT*, *CHI*, and *F3GT*). Among the structural genes, only the expression profile of *PsCHI* in “High Noon” was consistent with the accumulation pattern of total flavones/flavonols and faultily complementary to the expression pattern of novel_miR_138. The maximum expression of *PsCHI* was always one period ahead of the peaked accumulation of total flavones/flavonols (Figures 2, 3), indicating that novel_miR_138-*PsCHI* pair might affect the yellow coloring of “High Noon” flowers. Similarly, there are many cases of miRNAs directly targeting structural genes in flavonoid biosynthesis pathway, such as miR168 in canna directly targeting *CHS* (Roy et al., 2016), miR2616 and novel-miR25 in herbaceous peony regulating *F3GT* and *F3GT7*, respectively (Zhao et al., 2017a).

With respect to TFs, it is common knowledge that the roles of MYBs in flavonoid synthesis have been extensively investigated in plants (Stracke et al., 2007; Xu et al., 2015; Gu et al., 2018). In Arabidopsis, the TFs AtMYB11, AtMYB12, AtMYB111 activate early structural genes in flavonoid biosynthesis pathway such as *AtCHS*, *AtCHI*, and *AtF3H*, while AtPAP1, AtPAP2, AtMYB113, AtMYB114, and AtTT2 combine with AtTT8 to activate later structural genes (Stracke et al., 2007; Xu et al., 2015). Overexpression of *GhMYB1a* in *Gerbera hybrida* and *N. tabacum* inhibits anthocyanin accumulation, and promotes flavonol accumulation by up-regulating the corresponding structural genes related to flavonol biosynthesis (Zhong et al., 2020). In “High Noon,” the expression profile of novel_miR_260 was in accordance with the total flavone/flavonol accumulation trend, and its target gene *PsMYB1* showed an imperfect complementary expression pattern (Figures 2, 3). Therefore, it is speculated that novel_miR_260-*PsMYB1* module may play a critical role in yellow pigmentation of “High Noon” petals. Recently, WRKY TFs have also been proved to participate in flavonoid biosynthesis. PhPH3, a member of WRKY family in petunia, is responsible for the change of petal color by serving as a downstream regulator of the MBW complex (Verweij et al., 2016), and its homologous gene *VvWRKY26* in *V. vinifera* contributes to flavonoid accumulation (Amato et al., 2016). Moreover, PyWRKY26 interacts with PybHLH3, both of which co-targetes the *PyMYB114* promoter to positively regulate anthocyanin biosynthesis in red-skinned pear (Li et al., 2020). The expression pattern of *PsWRKY2* in “High Noon” was similar to that of total flavone/flavonol accumulation, whereas its regulator novel_miR_21 did not show a complementary expression pattern (Figures 2, 3). A similar case occurred in *PsNAC1* and its regulator novel_miR_101, and their regulatory relationship remains to be further studied. Nevertheless, NAC TFs play an irreplaceable role in flavonoid biosynthesis of other species. Apple NAC TF MdNAC52 regulates anthocyanin and proanthocyanin biosynthesis by binding to the promoters of *MdMYB9* and *MdMYB11* (Sun et al., 2019). The NAC TF, designated BLOOD (BL) in *Prunus persica*, acts with PpNAC1 to activate the expression of *PpMYB10.1*, while their transactivation activities are inhibited when the repressor *PpSPL1* exists (Zhou et al., 2015). It is particularly worth noting that the regulation modules between miR156 and SPL TFs have been reported to be involved in many plant physiological processes. For example, the mutation of OsmiR156 target site in *OsSPL14* decreases the tiller number and increases the grain yield of rice (Jiao et al., 2010). In maize, the miR156-*SPL* module in the drought-sensitive line is characteristically responsive to drought, accompanied by the opposite expression profiles in leaves and roots (Liu et al., 2019). Interestingly, in our study, the miR156-*SPL* pair was found to participate in the pigmentation. The expression pattern of *PsSPL2* was perfectly complementary to that of mdm-miR156b, and meanwhile negatively correlated with the accumulation pattern of total anthocyanins and positively correlated with the accumulation pattern of total flavones/flavonols. The *SPL9* targeted by miR156 in Arabidopsis also inhibits anthocyanin accumulation by directly down-regulating expression of structural genes in anthocyanin biosynthesis pathway (Gou et al., 2011). In *P. lactiflora*, the yellow formation of inner-petal might be under the regulation of miR156e-3p-targeted *SPL1* (Zhao et al., 2017b).

In order to further validate the potential function of *PsSPL2*, its full length sequence was isolated. Phylogenetic analysis showed that PsSPL2 was highly close to LcSPL1 (Figure 5A), which has been revealed to interact with LcMYB1 to regulate anthocyanin accumulation as a target gene of miR156a in *L. chinensis* (Liu et al., 2017). In our study, the reduction of anthocyanin content caused by *PsSPL2* overexpression in tobacco and the down-regulation of THC, Ap, and Ch content caused by *PsSPL2* silenting in tree peony were consistent, suggesting that *PsSPL2* probably played a crucial role in the yellow pigmentation of tree peony. Likewise, when SPL levels were reduced in miR156-overexpressing Arabidopsis plants, the levels of flavonols fell (Gou et al., 2011). Several *SPL*s targeted by miR156 such as *SPL1* in peach, *SPL2-like* and *SPL33* in apple, *SPL8* and *SPL13B* in pear have been proven to participate in the production of anthocyanin in fruits (Zhou et al., 2015; Qian et al., 2017; Yang et al., 2019). These findings support a notion that the regulation of miR156-SPL modules in flavonoid accumulation might be a conserved function in different plant species. Furthermore, we believed that *PsCHS*, *PsCHI*, and *PsF3H* might be responsible for the decreased production of THC, Ap, and Ch. In Arabidopsis, *CHS* and *CHI* also showed lower transcript levels in miR156-overexpressing plants (Gou et al., 2011). *VcSPL12* of *Vaccinium corymbosum* significantly reduces anthocyanin accumulation in Arabidopsis by repressing the expression of *AtTT7*, *AtPAP1*, *AtF3′H*, *AtDFR*, and *AtANS*. However, *VcMIR156a* greatly enhances anthocyanin production in *Solanum lycopersicum* by regulating several structural genes, especially *SlDFR* and *SlF3′5′H* (Li X. et al., 2020). When miR156e-3p of *P. lactiflora* was overexpressed in Arabidopsis, *SPL1* displayed significant down-regulation and *DFR* was strongly expressed, leading to an increased anthocyanin accumulation of lateral branches (Zhao et al., 2017b). In the present study, the significantly down-regulated expression of *NtDFR* in tobacco and up-regulated expression of *PsDFR* in tree peony also attracted our attention. It has been also reported that *DFR* transcripts were greatly increased, by over 30-fold, when *SPL* was inhibited in Arabidopsis (Gou et al., 2011). Taken together, there may be a complex regulatory network controlled by mdm-miR156b-PsSPL2 module to affect yellow pigment formation in tree peony flowers, and we will explore it in the future study. This study improved our understanding on miRNA-guided regulatory mechanism of flavonoid metabolism pathway in tree peony and would further help to elucidate the physiological process of yellow flower formation in tree peony.

## Data Availability Statement

The original contributions presented in the study are publicly available. This data can be found in the Sequence Read Archive of NCBI, accession number PRJNA763093.

## Author Contributions

XL, QS, and YZ conceived the research, designed the experiments, and wrote the manuscript. XL, SL, YF, CK, and KW performed the experiments. XL and DS analyzed the results. ML and ZY modified the language of the manuscript. All authors contributed to the article and approved the submitted version.

## Conflict of Interest

The authors declare that the research was conducted in the absence of any commercial or financial relationships that could be construed as a potential conflict of interest.

## Publisher’s Note

All claims expressed in this article are solely those of the authors and do not necessarily represent those of their affiliated organizations, or those of the publisher, the editors and the reviewers. Any product that may be evaluated in this article, or claim that may be made by its manufacturer, is not guaranteed or endorsed by the publisher.

## Footnotes

1
http://lowelab.ucsc.edu/GtRNAdb

2
http://www.arb-silva.de

3
http://www.girinst.org/repbase

4
http://rfam.xfam.org

5
https://db.cngb.org/search/project/CNP0000281/

6
http://www.australianprostatecentre.org/research/software/mirdeep-star

7
http://www.mirbase.org

8
ftp://ftp.ncbi.nih.gov/blast/db/

9
http://pfam.xfam.org/

10
http://www.uniprot.org/

11
http://bioinfo.cau.edu.cn/agriGO/analysis.php

12
https://www.kegg.jp/
