# Supplementary material for: Genome-Wide Identification and Comparative Profiling of MicroRNAs Reveal Flavonoid Biosynthesis in Two Contrasting Flower Color Cultivars of Tree Peony
Source: Front Plant Sci. 2022 Jan 4;12:797799. doi: 10.3389/fpls.2021.797799 (PMC8763678; doi:10.3389/fpls.2021.797799)
Supplement: Supplementary file 1 [file Data_Sheet_1.doc]

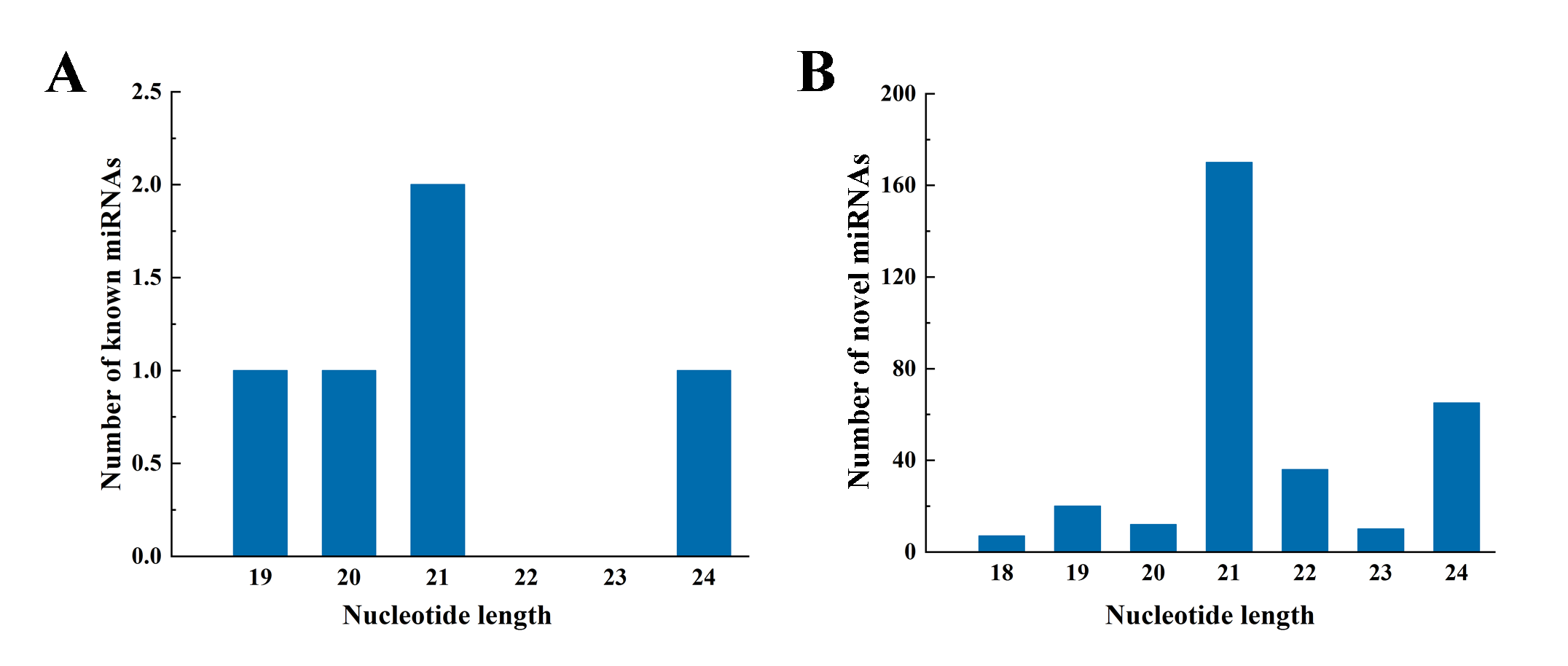


**Supplementary Figure 1** Length distribution diagram of the known **(A)** and novel **(B)** miRNAs.


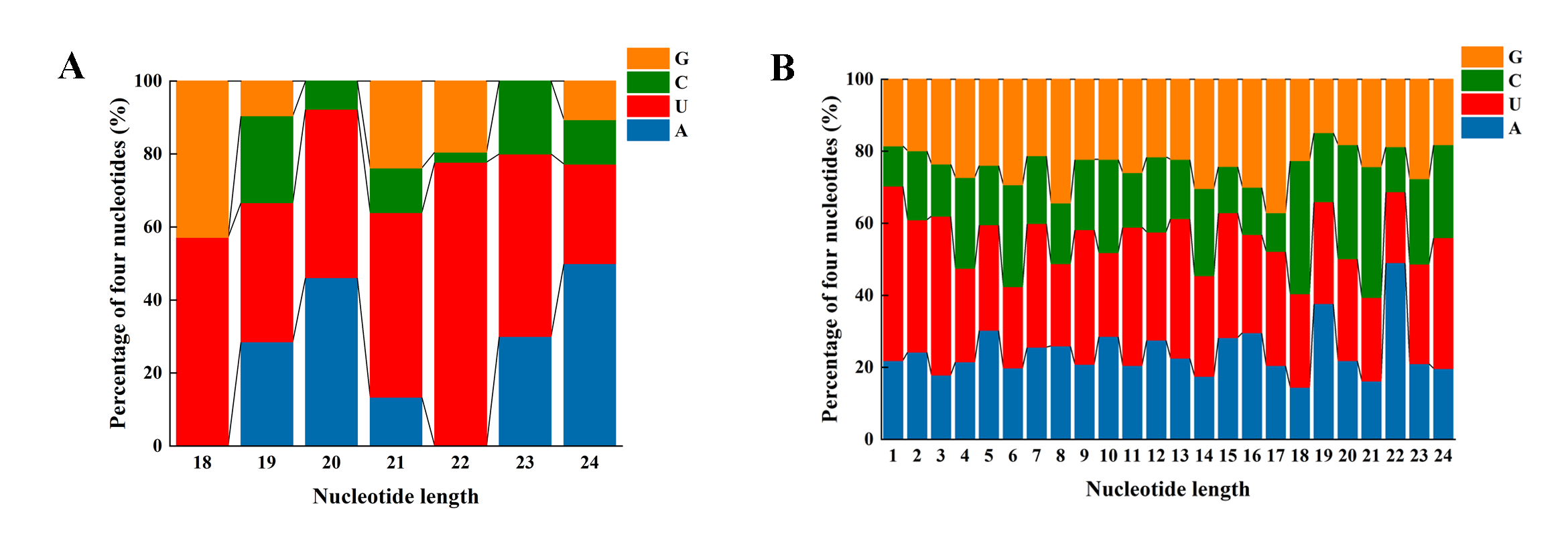


**Supplementary Figure 2** Analysis of miRNAs nucleotide bias. **(A)** Analysis of first nucleotide bias of miRNAs. **(B)** Analysis of each nucleotide bias of miRNAs.


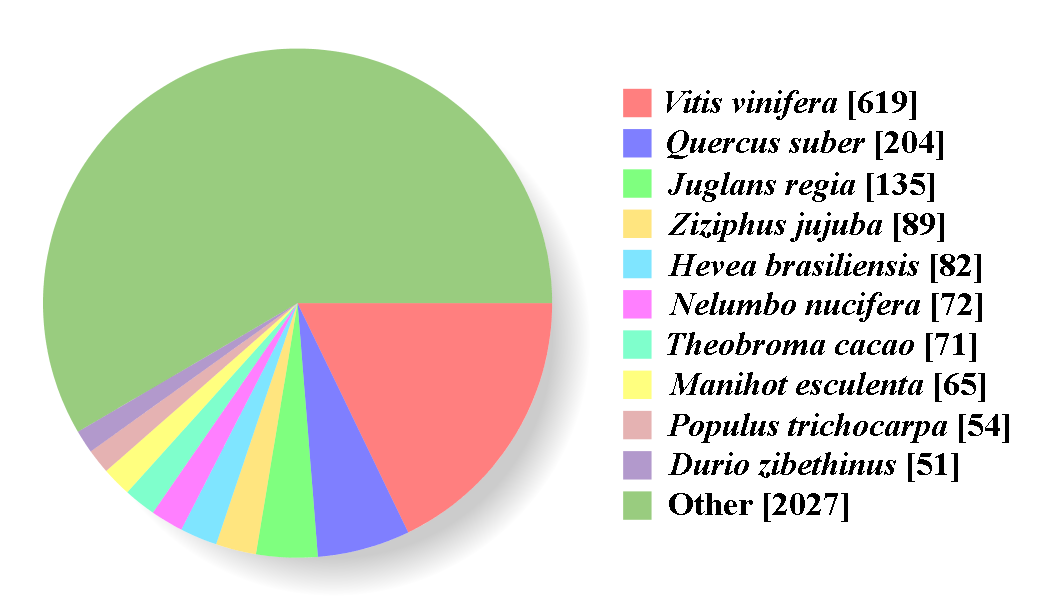


**Supplementary Figure 3** Nr Homologous species distribution diagram of target genes.


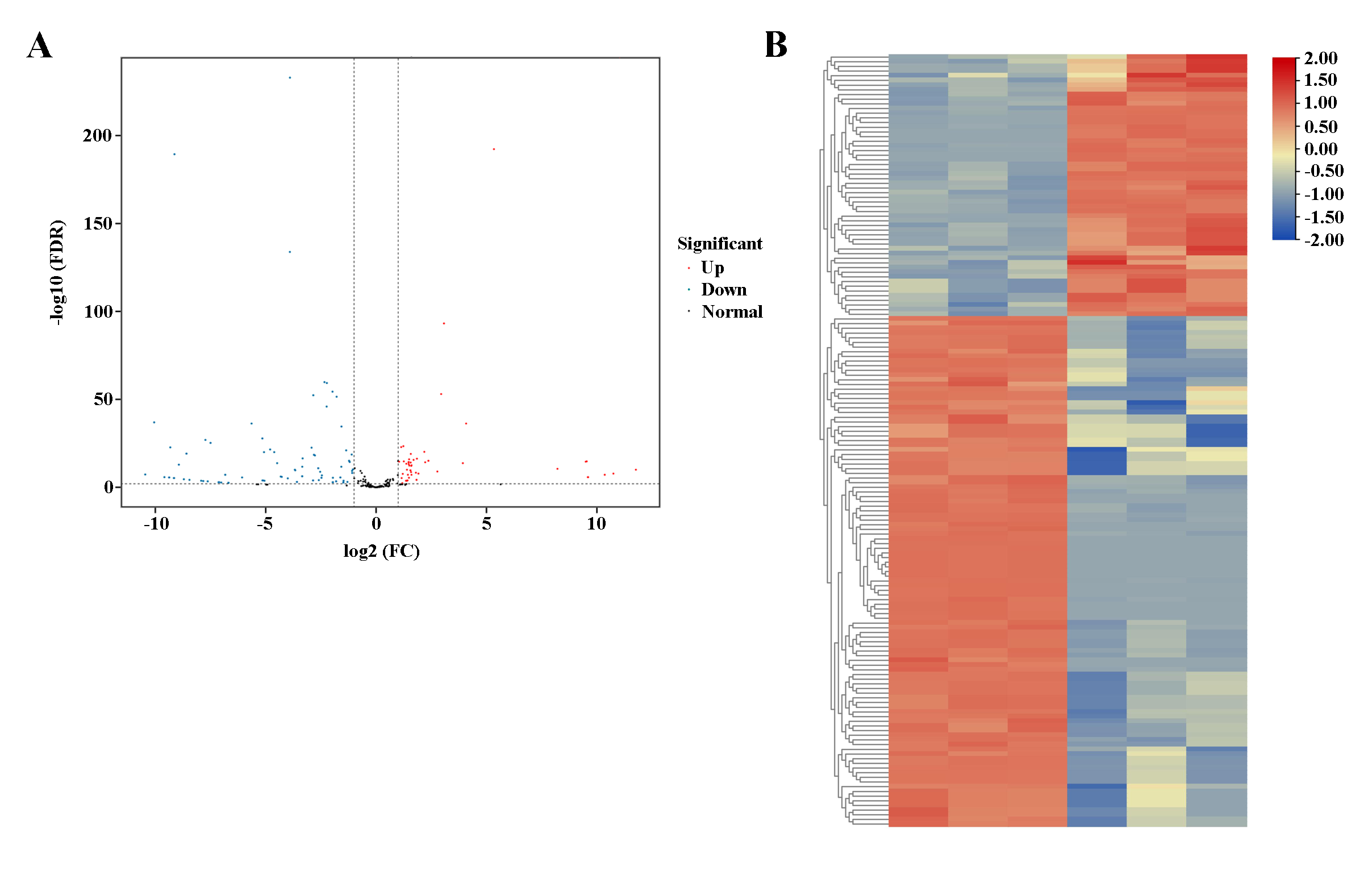


**Supplementary Figure 4 Analysis of differentially expressed miRNAs (DEMs) in ‘High Noon’ and ‘Roufurong’ libraries.** **A** Volcano plot of DEMs. **B** Cluster dendrogram of DEMs.


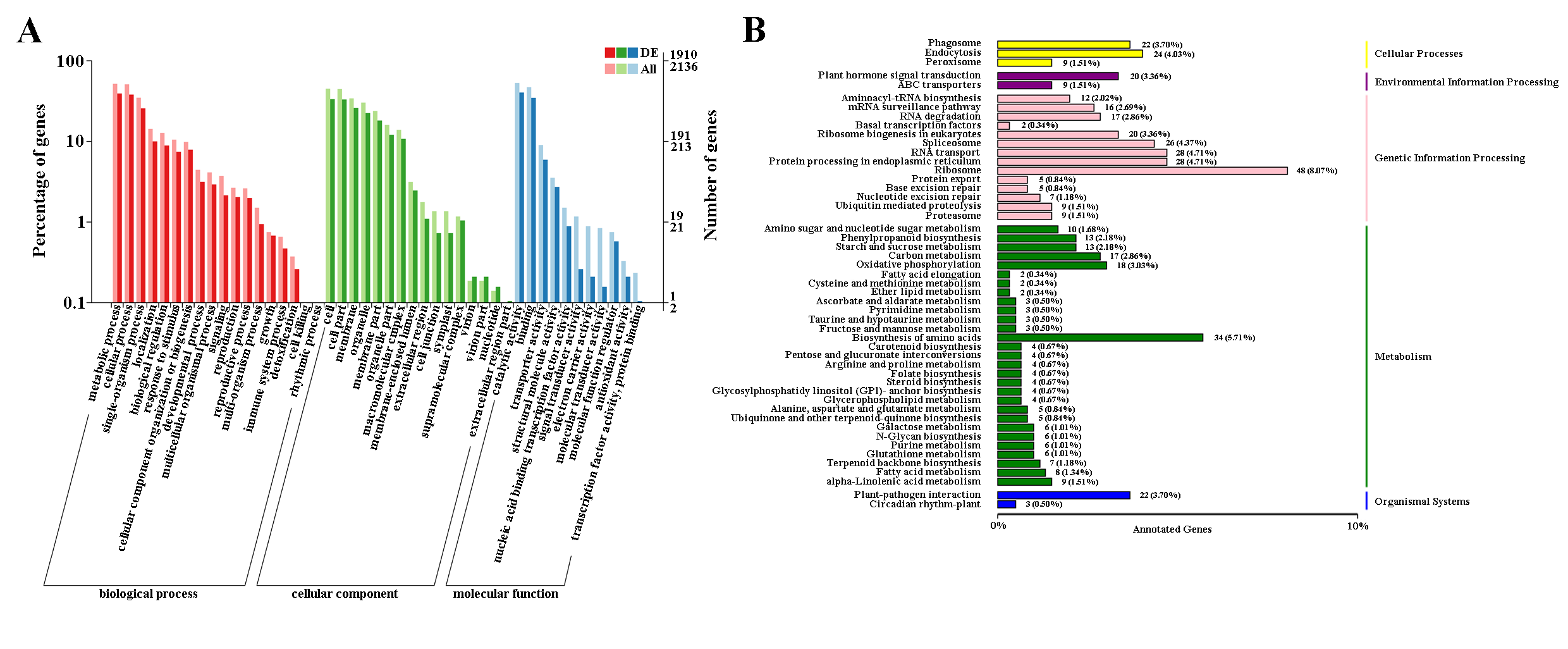


**Supplementary Figure 5 Functional annotation of differentially expressed miRNAs (DEMs) in ‘High Noon’ and ‘Roufurong’.** **A** GO enrichment analysis of DEMs. **B** KEGG enrichment analysis of DEMs.


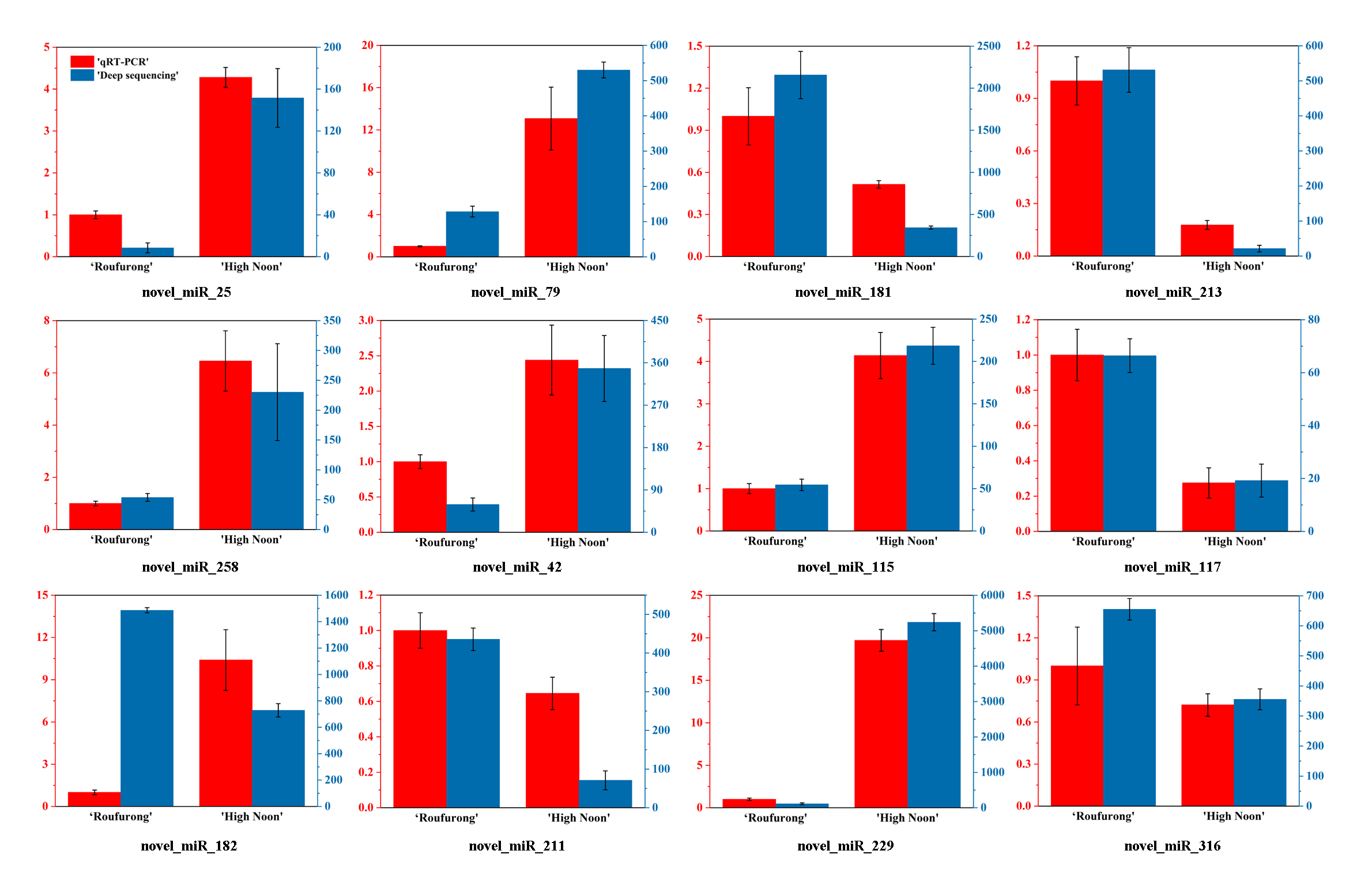


**Supplementary Figure 6** Comparison of the miRNA expression levels determined by deep sequencing and qRT-PCR. Blue and red colors indicate Transcript Per Million (TPM) and relative expression level obtained by deep sequencing and qRT-PCR, respectively. The error bars indicate the standard deviation obtained from biological replicates.
