# Supplementary material for: Genome-Wide Identification and Comparative Profiling of MicroRNAs Reveal Flavonoid Biosynthesis in Two Contrasting Flower Color Cultivars of Tree Peony
Source: Front Plant Sci. 2022 Jan 4;12:797799. doi: 10.3389/fpls.2021.797799 (PMC8763678; doi:10.3389/fpls.2021.797799)
Supplement: Supplementary file 2 [file Data_Sheet_2.doc]

| **Supplementary Table 1 Primers used for qRT-PCR and cloning in tree peony and tobacco.** | | |
| --- | --- | --- |
| **Primers** | **Primer Sequences (5'-3')** | **Description** |
| novel_miR_211-F | CTGAAGTGTTTGGGGGAACTC | qRT-PCR |
| novel_miR_182-F | TTTGTGCACCTCAGGCTCTTG | qRT-PCR |
| novel_miR_117-F | CTAGGGCATTATATTTAGGACC | qRT-PCR |
| novel_miR_316-F | TTTCGCCCTTTGTAACTTTCC | qRT-PCR |
| novel_miR_42-F | CACTTCAAACTATGCTCGACTT | qRT-PCR |
| novel_miR_115-F | ACAGTAGTCTGCACATTGGTT | qRT-PCR |
| novel_miR_229-F | TGGGATCAGAAGCTTGGCACT | qRT-PCR |
| novel_miR_25-F | TTTTTCGAACTGCAGGGGTGC | qRT-PCR |
| novel_miR_181-F | TTTCGCTCTTCATCTTCTGGA | qRT-PCR |
| novel_miR_213-F | TACTTGGACCCTGAATGAAGA | qRT-PCR |
| novel_miR_260-F | CGCATGTACTATGAATTGAGG | qRT-PCR |
| novel_miR_101-F | CGGTATAAAACTGTTGAAAATG | qRT-PCR |
| novel_miR_186-F | ATCTTCAGTGCTAATGTCTGG | qRT-PCR |
| mdm-miR156b-F | TGACAGAAGAGAGTGAGCAC | qRT-PCR |
| novel_miR_29-F | TCCCCTGCATCTCCACCG | qRT-PCR |
| novel_miR_21-F | CGTTTAAGGGATTTTAAAACATC | qRT-PCR |
| novel_miR_196-F | CGATTGATCCGACTTAAACGA | qRT-PCR |
| novel_miR_138-F | CGAGGAAGAAAGTAGTAGATGA | qRT-PCR |
| novel_miR_165-F | TTCCTGGATTTGGTTCTCGC | qRT-PCR |
| novel_miR_258-F | GGGTGGACTGCTCGAGCT | qRT-PCR |
| novel_miR_79-F | CGTTGAACACGAACTGGGAT | qRT-PCR |
| PsU6-F | CATCCGATAAAATTGGAACGA | qRT-PCR |
| PsU6-R | TTTGTGCGTGTCATCCTTGCG | qRT-PCR |
| PsMYB1(psu.G.00032738)-F | TTGCTGGTTTGGGGAGTGATT | qRT-PCR |
| PsMYB1(psu.G.00032738)-R | CGGTATTCAATCTCCCAAGCA | qRT-PCR |
| PsMYB2(psu.G.00021962)-F | GGGCAAATGTCTTCTCTCAGT | qRT-PCR |
| PsMYB2(psu.G.00021962)-R | GGAGATTCAAAGCTAAAGAGG | qRT-PCR |
| PsWRKY1(psu.G.00003037)-F | GGCGATGAACAAAGGCTGAAA | qRT-PCR |
| PsWRKY1(psu.G.00003037)-R | TTTTCTGCCCATATTTGCGCC | qRT-PCR |
| PsWRKY2(psu.G.00018110)-F | TTCTATTCTTTCCCACCTCCC | qRT-PCR |
| PsWRKY2(psu.G.00018110)-R | CATTCATTCAACTCCCCACAC | qRT-PCR |
| PsSPL1(psu.G.00031695)-F | CTTTACAACATCCACTCACCA | qRT-PCR |
| PsSPL1(psu.G.00031695)-R | GGAGCTGAGATCAAGAAATGG | qRT-PCR |
| PsSPL2(psu.G.00001221)-F | TCGGTTTGTGCTCTCTCTCTT | qRT-PCR |
| PsSPL2(psu.G.00001221)-R | GACATTGCTGACATTGGAAAC | qRT-PCR |
| PsSPL3(psu.G.00034722)-F | AAGAAAGCCTCAACAACCACC | qRT-PCR |
| PsSPL3(psu.G.00034722)-R | TTGTACGCTTGCCCATCTTGA | qRT-PCR |
| PsNAC1(psu.G.00032930)-F | TGAGCCTGTAGTTGATGATGA | qRT-PCR |
| PsNAC1(psu.G.00032930)-R | CTCTTCTTGACGGTAACACGG | qRT-PCR |
| PsNAC2(psu.G.00017706)-F | AATAAGCACCCAGAACAAGAG | qRT-PCR |
| PsNAC2(psu.G.00017706)-R | CTTCTCTTCTTCTTCCTCTTG | qRT-PCR |
| PsF5GT(psu.G.00004158)-F | CACGGATCACAAACTCTCACA | qRT-PCR |
| PsF5GT(psu.G.00004158)-R | CCTCGTTGTTGTAGTAGTAGT | qRT-PCR |
| PsCHI(psu.G.00026462)-F | AACAATGGAAGGGTAAAGCAG | qRT-PCR |
| PsCHI(psu.G.00026462)-R | TCATCGTATTTGTCATCGGCT | qRT-PCR |
| PsF3GT(psu.G.00030357)-F | TGTGATGTTTGGAAGATTGGC | qRT-PCR |
| PsF3GT(psu.G.00030357)-R | CCCTTGCTTTAAATTCTTTAT | qRT-PCR |
| PsUbiquitin-F | GACCTATACCAAGCCGAAG | qRT-PCR |
| PsUbiquitin-R | CGTTCCAGCACCACAATC | qRT-PCR |
| PsSPL2(psu.G.00001221)-F | ATGGATAAGTGGAAGGATCTA | Gene cloning |
| PsSPL2(psu.G.00001221)-R | CTAACAAGGGAGGAGAAAACT | Gene cloning |
| PsSPL2(psu.G.00001221)-F | ACACGGGGGACTCTTGACATGGATAAGTGGAAGGATCTA | In-fusion cloning |
| PsSPL2(psu.G.00001221)-R | AAGTTCTTCTCCTTTACTCTAACAAGGGAGGAGAAAACT | In-fusion cloning |
| TRV-PsSPL2-F | CCCATATGGTCGACCTGCAGTTTTGTGCAACTATCAAGGCA | In-fusion cloning |
| TRV-PsSPL2-R | GGACATGCCCGGGCCTCGAGAAGAGAGAGAGCACAAACCGA | In-fusion cloning |
| TRV-PsSPL2-F | TTGTTGATGGGTGTGATGCAG | qRT-PCR |
| TRV-PsSPL2-R | TTCTGGCTGAGGCTTTCTTCG | qRT-PCR |
| TRV-PsC4H-F | GTAATCTGGTGGTGGTTTCGT | qRT-PCR |
| TRV-PsC4H-R | TCGTAAAGAAAGGAACCGTCA | qRT-PCR |
| TRV-PsCHS-F | ACTACCAACTCACCAAACTCC | qRT-PCR |
| TRV-PsCHS-R | CGACCAAAGAATCCAAATGAG | qRT-PCR |
| TRV-PsCHI-F | CGCAGGAGTAAGAGGTTTGGA | qRT-PCR |
| TRV-PsCHI-R | CTCTGAATATTGTTGGCCCGT | qRT-PCR |
| TRV-PsF3H-F | AAAACCCTTCAATCCAGCTTC | qRT-PCR |
| TRV-PsF3H-R | AAATCCCCCAGTCTTCACATG | qRT-PCR |
| TRV-PsF3’H-F | TTTTGTGATACTGCCCCCTCT | qRT-PCR |
| TRV-PsF3’H-R | TATTTAGTGCAAAGATGGCCC | qRT-PCR |
| TRV-PsFLS-F | CACTCTCAACCAAAACAATCC | qRT-PCR |
| TRV-PsFLS-R | CACTCTCAACCAAAACAATCC | qRT-PCR |
| TRV-PsDFR-F | GTTAATGGACTCTGGGTTTGA | qRT-PCR |
| TRV-PsDFR-R | ATCCCTTTCCTCGGCATGTTT | qRT-PCR |
| NtTubA1-F | CTCCTATGCTCCTGTCATTTC | qRT-PCR |
| NtTubA1-R | GGCGAGGATCACACTTAAC | qRT-PCR |
| NtC4H-F | ACAACAGAGAAAGGCGGGCAA | qRT-PCR |
| NtC4H-R | AGGGGCTTTACATGACACCAA | qRT-PCR |
| NtCHS-F | TGACACCCACTTGGATAGTTTAG | qRT-PCR |
| NtCHS-R | CGACCTCTGGAATTGGATCAG | qRT-PCR |
| NtCHI-F | CTTTTCTCGCCGCTAAATG | qRT-PCR |
| NtCHI-R | TTTCTGCCACCTTCTCTG | qRT-PCR |
| NtF3H-F | CAAGGCATGTGTGGATATGG | qRT-PCR |
| NtF3H-R | TGTGTCGTTTCAGTCCAAGG | qRT-PCR |
| NtF3’H-F | AGGCTCAACACTTCTCGT | qRT-PCR |
| NtF3’H-R | CATCAACTTTGGGCTTCT | qRT-PCR |
| NtFLS-F | GAACTTGAAGGGAAAAGGGGTTG | qRT-PCR |
| NtFLS-R | GTAGGAGGGAGGATTTTTAGGCC | qRT-PCR |
| NtDFR-F | AACCAACAGTCAGGGGAATG | qRT-PCR |
| NtDFR-R | TTGGACATCGACAGTTCCAG | qRT-PCR |

| **Supplementary Table 2 Data output statistics of small RNA (sRNA) sequences** | | | | | | | |
| --- | --- | --- | --- | --- | --- | --- | --- |
| **Samples** | **Raw reads** | **Low quality** | **Containing 'N' reads** | **Length＜18** | **Length＞30** | **Clean reads** | **Q30(%)** |
| High Noon'-1 | 17,290,667 | 0 | 0 | 4,147,277 | 1,380,788 | 11,762,602 | 99.27 |
| High Noon'-2 | 18,107,874 | 0 | 0 | 1,760,494 | 2,150,150 | 14,197,230 | 99.23 |
| High Noon'-3 | 19,682,500 | 0 | 0 | 1,916,082 | 3,022,344 | 14,744,074 | 99.25 |
| Roufurong'-1 | 21,028,772 | 0 | 0 | 2,528,562 | 2,145,364 | 16,354,846 | 98.21 |
| Roufurong'-2 | 19,120,017 | 0 | 0 | 2,414,948 | 1,459,358 | 15,245,711 | 99.25 |
| Roufurong'-3 | 20,590,060 | 0 | 0 | 2,380,304 | 1,472,120 | 16,737,636 | 99.32 |

| **Supplementary Table 3 Statistics of sRNA data mapped to the reference genome** | | | | |
| --- | --- | --- | --- | --- |
| **Samples** | **Total reads** | **Mapped reads** | **Mapped reads (+)** | **Mapped reads (-)** |
| High Noon'-1 | 6,373,831 | 2,267,280 (35.57%) | 1,851,122 (29.04%) | 416,158 (6.53%) |
| High Noon'-2 | 7,589,847 | 2,784,909 (36.69%) | 2,285,298 (30.11%) | 499,611 (6.58%) |
| High Noon'-3 | 7,591,068 | 2,779,648 (36.62%) | 2,282,827 (30.07%) | 496,821 (6.54%) |
| Roufurong'-1 | 13,267,621 | 7,446,564 (56.13%) | 6,015,479 (45.34%) | 1,431,085 (10.79%) |
| Roufurong'-2 | 12,332,297 | 6,968,934 (56.51%) | 5,629,747 (45.65%) | 1,339,187 (10.86%) |
| Roufurong'-3 | 13,676,325 | 7,689,437 (56.22%) | 6,201,661 (45.35%) | 1,487,776 (10.88%) |

Mapped reads (+): The number of clean reads mapped to the ‘+’ chain

Mapped reads (-): The number of clean reads mapped to the ‘-’ chain

**Supplementary Table 4 Known and novel miRNAs identified from six libraries**

| **Samples** | **Known miRNAs** | **Novel miRNAs** | **Total** |
| --- | --- | --- | --- |
| High Noon'-1 | 4 | 267 | 271 |
| High Noon'-2 | 4 | 272 | 276 |
| High Noon'-3 | 5 | 269 | 274 |
| Roufurong'-1 | 4 | 312 | 316 |
| Roufurong'-2 | 4 | 312 | 316 |
| Roufurong'-3 | 3 | 312 | 315 |
| Total | 5 | 320 | 325 |

**Supplementary Table 5 The number of predicted miRNA target genes**

| **Types** | **All miRNAs** | **miRNAs with targets** | **Target genes** |
| --- | --- | --- | --- |
| Known miRNAs | 5 | 5 | 245 |
| Novel miRNAs | 320 | 289 | 4583 |
| Total | 325 | 294 | 4771 |

**Supplementary Table 6 The results of target gene annotation**

| **Databases** | **Annotated number** | **300<=length<1000** | **Length>=1000** |
| --- | --- | --- | --- |
| COG | 1,165 | 277 | 882 |
| GO | 2,136 | 664 | 1,456 |
| KEGG | 1,594 | 515 | 1,063 |
| KOG | 2,955 | 760 | 2,174 |
| Pfam | 2,872 | 665 | 2,191 |
| Swiss-Prot | 2,668 | 831 | 1,814 |
| NR | 3,475 | 1,042 | 2,405 |
| Total | 4,532 | 1,194 | 3,303 |
